# Oropouche Fever Outbreak, Manaus, Brazil, 2007–2008

**DOI:** 10.3201/eid1512.090917

**Published:** 2009-12

**Authors:** Maria Paula G. Mourão, Michelle S. Bastos, João Bosco L. Gimaque, Bruno Rafaelle Mota, Giselle S. Souza, Gustavo Henrique N. Grimmer, Elizabeth S. Galusso, Eurico Arruda, Luiz Tadeu M. Figueiredo

**Affiliations:** Tropical Medicine Foundation of Amazonas State, Manaus, Brazil (M.P.G. Mourão, M.S. Bastos, J.B.L. Gimaque, E.S. Galusso); Amazonas State University, Manaus (M.P.G. Mourão, J.B.L. Gimaque, B.R. Mota); Nilton Lins University Center, Manaus (M.P.G. Mourão, E.S. Galusso); Leonidas and Maria Deane Research Center, Manaus (G.S. Souza, G.H.N. Grimmer); University of São Paulo School of Medicine, Ribeirão Preto, Brazil (E. Arruda, L.T.M. Figueiredo)

**Keywords:** Oropouche fever, arbovirus, vector-borne infections, letter

**To the Editor:** Oropouche virus (OROV) is an arbovirus, *Orthobunyavirus*, transmitted among sloths, marsupials, primates, and birds by the mosquitoes *Aedes serratus* and *Culex* *quinquefasciatus*. Notably, this virus has adapted to an urban cycle involving man, with midges (*Culicoides paraensis*) as the main vector (*1*). Oropouche fever is the second most frequent arboviral disease in Brazil, surpassed only by dengue. OROV causes large, explosive outbreaks of acute febrile illness in cities and villages in the Amazon and central regions of Brazil. An estimated 500,000 cases of OROV infection have occurred in Brazil in the past 48 years. In addition to outbreaks, OROV can also cause sporadic human infections (*2*).

The Tropical Medicine Foundation of Amazonas State (TMF-AM) is a tertiary care center specializing in tropical and infectious diseases and is located in the city of Manaus. Syndromic surveillance for acute febrile illness has been conducted by TMF-AM since 1998. During January 2007 through November 2008, we obtained blood samples from 631 patients who had acute febrile illness for ≥5 days but who had negative results at initial screening for malaria (thick blood smear) and dengue (MAC-ELISA). Blood samples were tested for OROV immunoglobulin (Ig) M antibodies by an indirect enzyme immune assay using infected cells as antigen, as previously reported for dengue (*3*).

For the indirect enzyme immune assay using infected cells as antigen, C6/36 *A. albopictus* cells were grown in 96 well microplates; these cells were infected with OROV (BeAn 1991 strain). After 4 days, the cells were fixed in the wells with 7% formalin buffered at pH 7.0. The microplate was blocked with 5% skim milk and, after washing the wells, 100 µL of serum diluted 1:400 was added into infected and uninfected wells. After incubation and washing the wells, a peroxidase-conjugated goat anti-human IgM was added; finally, the ABTS substrate (KPL, Inc., Gaithersburg, MD, USA) was added into the wells. The plates were incubated and read on a spectrophotometer at 405 nm. The cutoff for the test was determined to be the mean of optical densities read in all wells containing uninfected cells plus 3 standard deviations.

Of the 631 patients in the study, 128 (20.3%) had IgM antibodies to OROV. The age range was 2–81 years (mean 29.5 ± 14 years), and 77 (60.2%) were women or girls. Most of the cases occurred November through March during the rainy season. In addition to fever, the patients had headache (93 [72.7%]), myalgia (90 [70.3%]), and arthralgia (74 [57.8%]). Rash was observed in 54 patients (42.2%), and hemorrhagic phenomena (petechiae, epistaxis, and gingival bleeding) were observed in 20 patients (15.5%). All patients recovered without sequelae and were not hospitalized.

Despite the knowledge of the occurrence of several arboviruses in the Amazon region, most cases of arboviral diseases remain undiagnosed, probably because of their generally mild and self-limited clinical manifestations. Patients usually recover completely after a couple of days. However, even more severe cases may remain undiagnosed, especially because of long distances to health care facilities, difficulties in sample transportation, and lack of laboratory facilities capable of conducting the diagnostic assays. With regard to OROV infections, diagnosis of OROV may be easily confused with other acute febrile illness, including malaria and dengue, both of which are highly endemic in Manaus.

In the present study, an inhouse enzyme immune assay for IgM using infected cell culture as antigen was found suitable for the diagnosis of OROV infections in the acute phase. Thus, a combination of a systematic surveillance for acute febrile illnesses and efficient laboratory diagnosis for OROV resulted in the discovery of an outbreak, which would probably have been overlooked if it had occurred in any region simultaneously with large dengue outbreaks or in the absence of laboratory diagnosis. The cases of OROV fever reported here likely represent a small portion of the cases; a much higher number of cases probably occurred in Manaus during the study period.

The clinical characteristics of most cases of OROV fever in this outbreak were similar to previously reported descriptions of the illness. Notably, however, 20 (15.5%) patients from Manaus had spontaneous hemorrhagic phenomena (petecchiae, epistaxis, and gingival bleeding) that had not previously been described in OROV fever (*4*–*6*). Moreover, symptoms of involvement of the central nervous system were not observed.

In recent years, the area of circulation and the epidemic potential of OROV have increased, and this virus has emerged as a public health problem in Brazil and other countries in the Americas. Presently, OROV is the most common of the Brazilian zoonotic arboviruses infecting humans (*7*). Further evidence of the spread of OROV was its isolation in 2003 from a small primate, a marmoset (*Callithrix*), in the state of Minas Gerais in southeast Brazil, far from the Amazon region (*8*). Considering that midges (*Culicoides paraensis*) occur in most low altitude areas of the Americas, it is conceivable that environmental destruction and climate changes could result in OROV outbreaks in the large cities of Brazil, as well as in other parts of the Western Hemisphere (*9*).
